# Normalization of Prediabetic Hemoglobin A1c (HbA1c) Levels After the Surgical Removal of a Serotonin-Secreting Neuroendocrine Tumor

**DOI:** 10.7759/cureus.57376

**Published:** 2024-04-01

**Authors:** Aleksandre Tskitishvili, Mariam Lobjanidze, Zurabi Turmanishvili, Nikoloz Mamulashvili, Tamar Bejanishvili

**Affiliations:** 1 Aieti Medical School, David Tvildiani Medical University, Tbilisi, GEO; 2 Internal Medicine, Brookdale Hospital and Medical Center, Brooklyn, USA; 3 Primary Care, University Hospitals of Cleveland, Bedford, USA

**Keywords:** serotonin-secreting tumor, glycated hemoglobin (hba1c), functional neuroendocrine tumor, carcinoid, prediabetes regression

## Abstract

Neuroendocrine tumors (NETs) are rare. When present, they often produce serotonin and are called carcinoids. Serotonin-secreting NETs can present with or without carcinoid syndrome. Although the idea of serotonin-secreting NETs potentially altering glucose metabolism is not new, data around this issue has been scarce, with only a few limited studies and case reports. We present a case where a female patient’s prediabetic hemoglobin A1C levels normalized after removing serotonin-secreting NET. Before removal, the patient had locally metastatic carcinoid and serotonin-related intractable diarrhea but did not exhibit any other sign of carcinoid syndrome, including flushing, which is considered a hallmark. Therefore, in suggestive clinical contexts, this case points to the possibility of impaired glucose tolerance being an early clinical sign of carcinoid that could aid in serotonin-secreting NET diagnosis before it manifests as overt carcinoid syndrome.

## Introduction

Neuroendocrine tumors (NETs) are rare entities in internal medicine, constituting 0.5% of all cancer diagnoses [1,2]. Among them, carcinoid tumors are the most common, primarily found in the gastrointestinal tract. Due to their secretory nature, NETs can cause neuroendocrine substance-related symptoms that might long precede the development of mass effects. One such sign is impaired glucose tolerance (IGD). Although the idea that serotonin-secreting tumors could alter glucose metabolism has existed since the 1970s [3,4], there has been a scarcity of epidemiologic data about the frequency of this phenomenon. Connecting this metabolic disturbance to NETs causally may be very challenging as prediabetes is a much more common condition that may simply coexist with NETs without any pathophysiologic relationship [5]. We present the case of a 58-year-old female with prediabetes who had her hemoglobin A1c (HbA1c) normalized after surgical removal of serotonin-producing NET. To our knowledge, this is the first case report on this topic.

## Case presentation

A 58-year-old female with a history of intellectual disability, epilepsy, overweight, prediabetes, and chronic diarrhea came to the primary care clinic for a wellness examination. Despite the developmental delay, the patient was able to communicate clearly. Before this, the patient was seen by a gastroenterologist who ordered stool pathology and pathogen tests (samples were never collected), gastroscopy, and colonoscopy to determine the cause of the diarrhea. Her medications included metformin, aripiprazole, carbamazepine, trazodone, vitamin D, and multivitamin supplements.

During her first primary care visit, the patient reported fecal incontinence that caused multiple accidents per day. Physical examination revealed poor dentition with multiple loose and absent teeth but was otherwise normal, including the absence of weight loss, pruritus, abdominal pain, and nausea. Pulmonary and cardiovascular examinations were also unremarkable. The patient had a history of overweight and prediabetes and was taking metformin. Her body mass index on the visit was 30 kg/m^2^, and her HbA1c level was 6.3%. On this visit, dulaglutide was started, and metformin was stopped to assess the medication’s possible role in the patient’s diarrhea. However, the substitution did not change the frequency of the patient’s incontinence, and dulaglutide was discontinued within weeks due to uncomfortable side effects.

**Figure 1:**
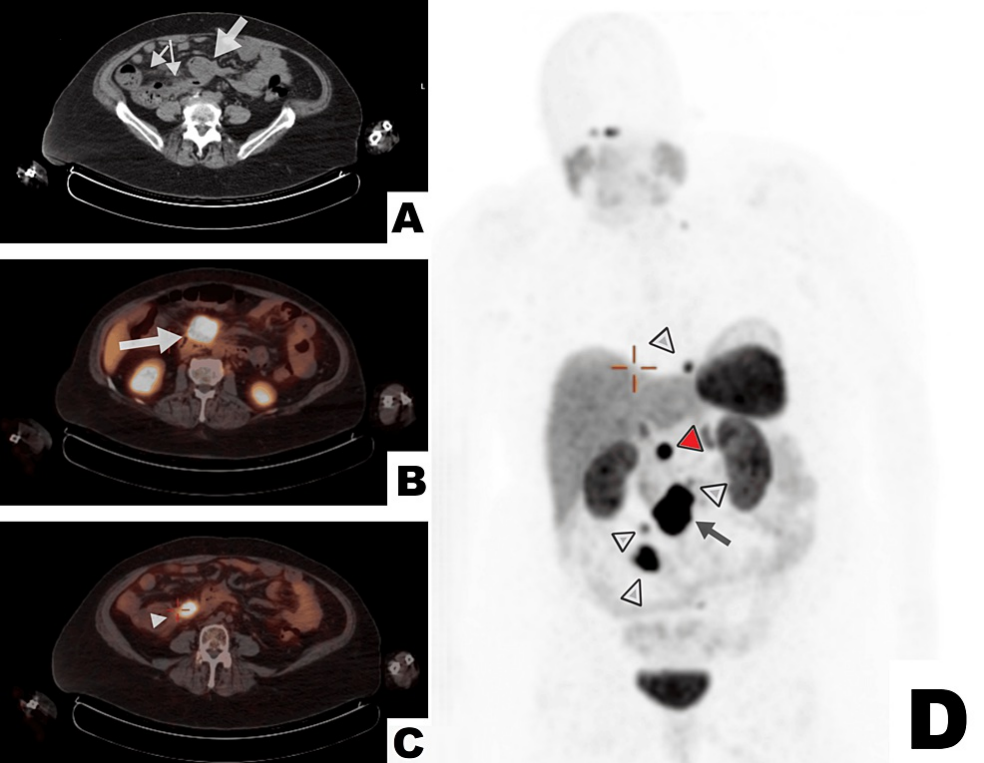
Imaging findings suggesting the diagnosis of a neuroendocrine tumor. A: A transverse CT section of the lower abdomen showing an irregular mass just anterior to the ileum (thick arrow) and thickening of the cecal and ileal walls (thinner arrows). B: Increased uptake of ^64^Cu-DOTATATE (arrow) by the malignant mass on positron emission tomography (PET) scan. C: Increased ^64^Cu-DOTATATE uptake (arrowhead) by a sizeable secondary lesion inferolateral to the primary on PET scan. D: Increased ^64^Cu-DOTATATE uptake in the primary lesion (arrow) and metastases (light arrowheads) on maximum intensity projection image of PET scan, coronal section. Note: The increased radiotracer uptake indicated by the red arrowhead represents the normal functioning of the uncinate process of the pancreas. The unlabeled opacities of the kidneys, liver, spleen, bladder, adrenal, salivary, and pituitary glands also represent the physiologic uptake.

Two weeks after the primary care visit, the patient underwent a colonoscopy that found a mass in the terminal ileum. A biopsy of the mass identified a well-differentiated NET, grade 1. Staining was positive for insulinoma-associated protein 1, chromogranin (INSM-1), caudal-type homeobox 2 (CDX-2), special AT-rich sequence-binding protein 2 (SATB-2), and serotonin, and the Ki-67 index was 0.6%. To assess the extent of the disease, CT of the chest, abdomen, and pelvis was performed, revealing a 5.3 cm mesenteric mass in the right hemiabdomen with numerous prominent surrounding nodes and wall thickening of the ileum and cecum (Figure 1A). No liver or thoracic metastases were detected. The tumor was further characterized by a ^64^Cu-DOTATATE positron emission tomography (PET) scan that showed a somatostatin receptor-positive lesion in the terminal ileum (Figure 1B) and metastases (Figures 1C, 1D) involving numerous surrounding lymph nodes.

Laparoscopy was started and converted to open ileocolectomy. The mesenteric mass, middle colic lymph node, and mesorectal nodule were resected, and an ileocolic anastomosis was made. Pathologic examination showed a well-differentiated G1-2 tumor with regional lymph node metastases, corresponding to the T3N2 stage by the American Joint Committee on Cancer classification, eighth edition. Local fibrous adhesions were detected between the ileum and cecum but not elsewhere.

On the subsequent primary care visit 3.5 weeks after the surgery, the patient reported one to two loose bowel movements daily. Her caregiver denoted a single flushing episode with periorbital swelling shortly after her surgery. The patient had been consuming her usual diet, doing all her regular activities, and having no other complaints. Upon the patient’s last visit two more weeks later, bowel movements were already standard. Upon testing, urinary 5-hydroxyindoleacetic acid (5-HIAA) was down from 66 to 38 μmol/day. Her HbA1c level had decreased to 5.0% despite the patient not receiving any diabetes medication for the last three months and consuming her usual diet.

## Discussion

The dramatic improvement in HbA1c levels in this case points to the possible causal relationship between serotonin-secreting NET and IGD. Although there is a theoretical possibility this tumor could also have produced additional substances that could elevate glucose levels, the probability of this is very slim with gastrointestinal NETs [6]. The idea of serotonin causing IGD was first explored in the 1970s, with the serotonin-mediated decrease in insulin production being put forward as a possible explanation [4,7,8]. However, half a century later, the exact mechanism of serotonin’s action on insulin levels still needs to be clarified and is thought to be exceedingly complex [9-11]. The sample size of early studies that studied the link between serotonin-secreting NETs and IGD was limited (fewer than 20 patients) [3,4], and there has been an appreciable scarcity of epidemiologic frequency data or even case reports regarding this causal interaction ever since.

Carcinoid syndrome complicates as much as 55.5% of advanced well-differentiated NETs originally derived from small bowel and is associated with decreased survival [12]. Notably, the patient’s prediabetes occurred in the absence of flushing (a surgical substance leak presumably caused the single flushing episode after the surgery), widely regarded as a hallmark of carcinoid syndrome, or other relatively common manifestations such as abdominal pain, cardiac valve involvement, or bronchospasm [13,14]. Therefore, IGD might serve as an earlier clinical sign of serotonin-secreting NETs. As noted in the introduction section, IGD and prediabetes are much more common than NETs, and these conditions can often merely coexist, making causal inference very challenging. However, if IGD occurs in a suggestive clinical context that comprises some signs of (for example, as in this case, intractable diarrhea) but does not yet suggest carcinoid syndrome, it could serve as an additional clinical clue and allow putting serotonin-secreting NET higher in the list of suspected conditions. Still, in the end, considering the paucity of data on this subject, further studies are required to determine the presence and strength of the causal relationship between serotonin-secreting NETs, IGD, and prediabetes.

## Conclusions

This case report adds to the scarce evidence regarding the possible causal link between serotonin-secreting NETs and IGD. It also points to the possibility of elevated serotonin impairing glucose tolerance without presenting as overt carcinoid syndrome. Therefore, in suggestive clinical contexts, IGD may serve as a potential additional early clinical clue for the presence of and assessment for serotonin-secreting NET when it does not or before it causes carcinoid syndrome. Further research may allow a better description of this possible causality between serotonin-secreting NETs, IGD, and prediabetes.
